# FeetBack–Redirecting touch sensation from a prosthetic hand to the human foot

**DOI:** 10.3389/fnins.2022.1019880

**Published:** 2022-10-26

**Authors:** Rafael Morand, Tobia Brusa, Nina Schnüriger, Sabrina Catanzaro, Martin Berli, Volker M. Koch

**Affiliations:** ^1^Biomedical Engineering Lab, Institute for Human Centered Engineering, Bern University of Applied Sciences, Bern, Switzerland; ^2^Division of Prosthetics and Orthotics, Department of Orthopedics, Balgrist University Hospital, Zurich, Switzerland

**Keywords:** upper limb prosthesis, sensory feedback, touch sensation, grip force, vibrotactile insole, discrete feedback

## Abstract

**Introduction:**

Adding sensory feedback to myoelectric prosthetic hands was shown to enhance the user experience in terms of controllability and device embodiment. Often this is realized non-invasively by adding devices, such as actuators or electrodes, within the prosthetic shaft to deliver the desired feedback. However, adding a feedback system in the socket adds more weight, steals valuable space, and may interfere with myoelectric signals. To circumvent said drawbacks we tested for the first time if force feedback from a prosthetic hand could be redirected to another similarly sensitive part of the body: the foot.

**Methods:**

We developed a vibrotactile insole that vibrates depending on the sensed force on the prosthetic fingers. This self-controlled clinical pilot trial included four experienced users of myoelectric prostheses. The participants solved two types of tasks with the artificial hands: 1) sorting objects depending on their plasticity with the feedback insole but without audio-visual feedback, and 2) manipulating fragile, heavy, and delicate objects with and without the feedback insole. The sorting task was evaluated with Goodman-Kruskal's gamma for ranked correlation. The manipulation tasks were assessed by the success rate.

**Results:**

The results from the sorting task with vibrotactile feedback showed a substantial positive effect. The success rates for manipulation tasks with fragile and heavy objects were high under both conditions (feedback on or off, respectively). The manipulation task with delicate objects revealed inferior success with feedback in three of four participants.

**Conclusion:**

We introduced a novel approach to touch sensation in myoelectric prostheses. The results for the sorting task and the manipulation tasks diverged. This is likely linked to the availability of various feedback sources. Our results for redirected feedback to the feet fall in line with previous similar studies that applied feedback to the residual arm.

**Clinical trial registration:**

Name: Sensor Glove and Non-Invasive Vibrotactile Feedback Insole to Improve Hand Prostheses Functions and Embodiment (FeetBack). Date of registration: 23 April 2019. Date the first participant was enrolled: 3 September 2021. ClinicalTrials.gov Identifier: NCT03924310.

## 1. Introduction

The human hand allows us to explore the environment by touch sensation such that we feel the temperature, texture, and applied force. Myoelectric control for prosthetic hands advanced in terms of dexterity and allows for complex grip patterns. However, there is no commercially available system with sophisticated touch sensation^1^. There are only a few commercially available hands that provide information about initial contact and object release. Even less devices provide information about the grip force *via* vibrations within the socket of the prosthesis. Yet, adding touch sensation to prosthetic hands has been an ongoing topic in research in the past decades (Antfolk et al., 2013; Sensinger and Dosen, 2020). The motivation behind adding touch sensation lies in improving the system control and strengthening the user's feeling of agency and body ownership. Furthermore, the lack of sensory feedback in prostheses is a leading cause of device abandonment among other functional issues (difficult to control, slow response speed, poor dexterity) and comfort issues (temperature, weight, poor fit) (Smail et al., 2021; Jabban et al., 2022). The information from touch sensation that is commonly fed back are the grip force (or individual finger force), proprioception of the hand aperture, initial contact and object release, and grip selection (for hands with multiple degrees of freedom). Various approaches have been tried which can be divided into two categories: invasive and non-invasive methods. Invasive methods feed back the touch information through implanted electrodes that directly stimulate the nerves. Such methods achieve remarkable results with respect to user acceptance and improvement of control (Graczyk et al., 2018; Schiefer et al., 2018; D'Anna et al., 2019). However, improvements come with risks associated with surgery. Alternatively, information from touch sensation can be delivered with non-invasive methods. Commonly used channels are of mechanotactile, vibrotactile or electrotactile nature (Stephens-Fripp et al., 2018; Masteller et al., 2021). Vibrotactile systems employ vibrational motors and are perceived by receptors in the skin. They can be used to transmit information by varying the stimulation amplitude, frequency, duration, and shape. Vibration is used substitutionary when the source of the feedback is pressure, e.g., on the fingers of the prosthetic hand. Vibrotactile systems were shown to allow for simple to interpret signals (Stephens-Fripp et al., 2018). However, they introduce a short delay due to the ramp-up time and are limited in the bandwidth which in turn limits the capacity to transmit information. Electrotactile systems stimulate cutaneous fibers. This allows transmitting the sensations of vibration and pressure (Kaczmarek, 2000). Recent advances in electrotactile feedback showed promising results for intuitive non-invasive feedback. Gholinezhad et al. (2021) reported that the participants' central nervous system could adopt the feedback subconsciously within a training time of less than 5 min. However, the perception depends heavily on the user it is applied to and the minute conditions of the skin (e.g., sweat). Thus, frequent readjustment of the stimulation parameters is required (Stephens-Fripp et al., 2018). Mechanotactile systems employ tactors to deliver modality matched sensations of pressure. However, the used tactors are often too bulky and energy demanding for portable systems (Antfolk et al., 2013). Many sources state the obvious benefits of touch feedback (Sensinger and Dosen, 2020). Furthermore, users rank the addition of touch feedback to myoelectric hand prostheses as a top priority (Wijk and Carlsson, 2015). Nevertheless, there is no consensus on the actual benefit of non-invasive feedback in clinical applications outside the lab (Markovic and Schweisfurth, 2018; Wijk et al., 2020). The benefits were often shown under limitations such as obstruction of incidental feedback (vision, hearing, motor vibrations of the prostheses) or experimental tasks of routine grasping that could be executed by feedforward control alone (Sensinger and Dosen, 2020). Feedforward control is a crucial aspect of human motor control and it is governed by the individual's internal model and understanding of cause to effect (Engels et al., 2019; Sensinger and Dosen, 2020). The Feedforward control is subject to noise. Therefore, feedback sources are necessary to detect and correct mismatches between the outcome and the expectation. However, the internal model can be trained with feedback, e.g., learning how to use a prosthetic device under known circumstances as demonstrated with EMG biofeedback (Dosen et al., 2015; Schweisfurth et al., 2016). Another example was shown by Markovic and Schweisfurth (2018) where the participants learned the necessary feedforward control during routine tasks. Eventually, the participants performed equally well with and without feedback in the given tasks. However, inappropriate feedback strategies were found to degrade the internal model (Engels et al., 2019) and incidental feedback such as vision alone may also just outperform other strategies depending on the task (Wilke et al., 2019).

In our previous research, we tested various feedback strategies such as tactor based feedback, vibration with coin vibration motors, and combinations of both (Li et al., 2016; Huang et al., 2017). Furthermore, we tested different sites to redirect the feedback, i.e., the residual limb, the upper arm, or the contralateral hand. The focus lies in redirecting the feedback to the phantom map of amputees (Huang et al., 2018), where applicable. However, we found two main limitations to these approaches:

1. Only a few people have a phantom map which limits the application.
2. If present, phantom maps are often found on the residual limb. Redirecting the feedback to that region results in adding more devices to the already burdened limb, steals valuable space within the socket, and may interfere with surface electromyographic signals (sEMG) for myoelectric control.

Therefore, we searched for a different body part to which to apply the feedback. We settled for the sole of the feet because it is among the most sensitive parts of the human body (Kennedy and Inglis, 2002). Moreover, this answers the user need of reducing the weight and the complexity of the prosthetic socket (Jabban et al., 2022). To favor miniaturization, we chose to rely on vibrotactile information from coin vibration motors without additional tactors. These motors can be embedded within an insole which can be worn within a shoe. This adds a design benefit by hiding the device inside the shoe as opposed to wearing it openly, e.g., as a vibrational cuff around the upper arm or the shanks. The viability of such feedback was already shown in mobile robot control (Jones et al., 2020) and in supported navigation while walking (Velázquez et al., 2012; Meier et al., 2015). Importantly, neither research reported burdening of the participants by e.g., additional weight or induced gait disturbances. A similar approach (Sasaki et al., 2018) introduced body-worn robotic arms. These arms are piloted with the feet and the hands' touch sensation is fed back to the sole of the feet. However, the authors did not comment on the effect of the feedback and stated that they intended to improve it in a future step. In a preliminary trial, we tested three feedback settings:

1. Continuous feedback for each finger applied to the toes.
2. Continuous feedback from the grasp force applied to the pinkie toe.
3. Discrete feedback from the grasp force applied as a spatially coded ramp along the foot.

We observed fair results with the discrete feedback which was likely the easiest to interpret (Aboseria et al., 2018). Therefore, we settled for a spatially coded discrete feedback device with a continuous sensing device for FeetBack. The sensed modality is the grip force. Users ranked it the top priority for sensory feedback in surveys with over hundred participants (Lewis et al., 2012; Smither et al., In Press).

The goal of this pilot study was to test the applicability of discrete tactile feedback applied to the feet to partially emulate touch sensation, i.e., grip force, of a prosthetic hand. We did not test to emulate proprioception, although the presented device should allow for it (similarly to Štrbac et al., 2016). Thus, we first describe the FeetBack system followed by the study participants and the experimental tasks. Eventually, the results are presented and discussed.

## 2. Methods

### 2.1. FeetBack system

#### 2.1.1. System overview

**Figure 1:**
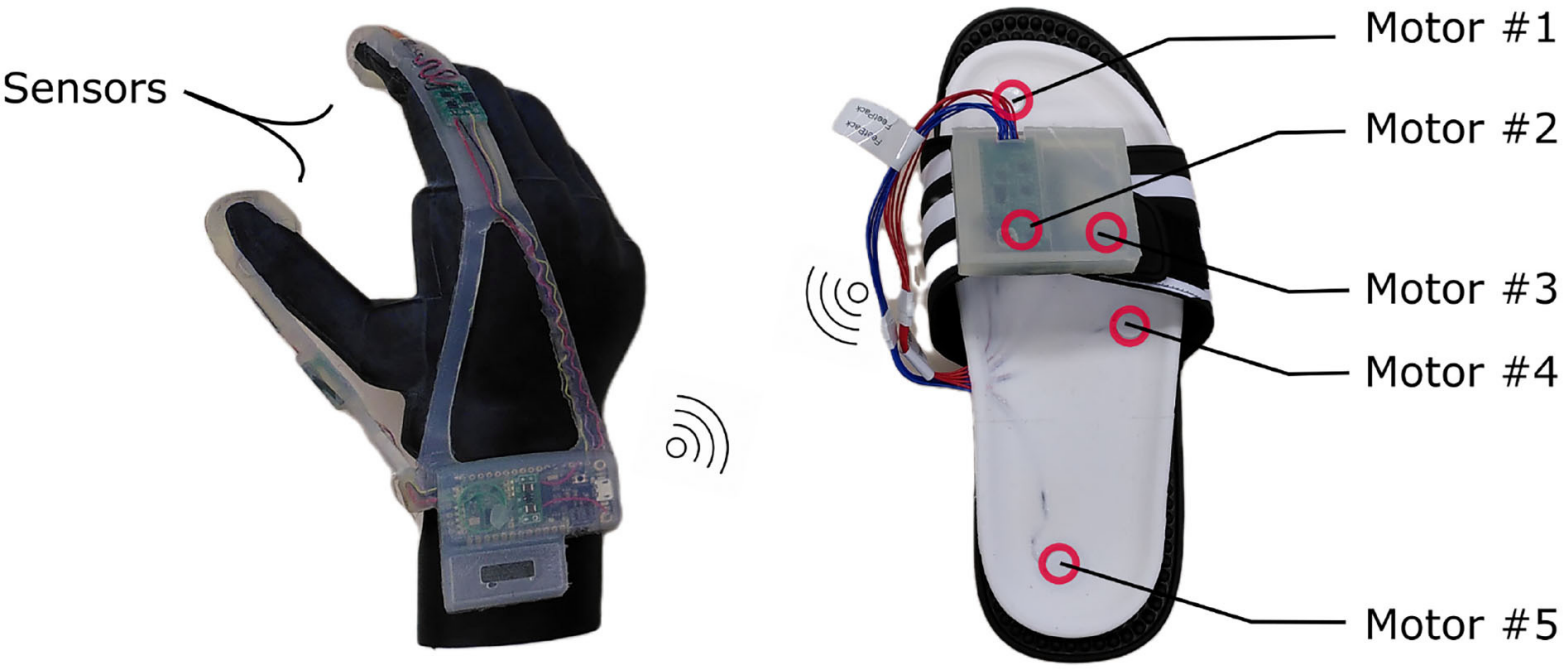
FeetBack glove and insole. The glove senses the pressure on the fingertips of the index finger and the thumb. The higher force at either finger is defined to represent the grip force and is sent wirelessly to the insole. Depending on the force one of the five embedded coin vibration motors starts vibrating. The lower the force, the lower the number of the running motor (or no motor at all when the force is below a minimal threshold). The distribution of the motors was chosen accordingly to the distribution of fast adapting mechanoreceptors on the sole of the foot.

The FeetBack system consists of a sensing glove and a feedback insole (Figure 1). The glove is equipped with one pressure sensor on the index finger and one on the thumb. The sensed force is sent wirelessly (Adafruit M0 RFM69 Packet Radio) to the insole, where the value is converted to a distinct force level. Depending on the force level, either none or one of five embedded vibration motors starts buzzing. The conversion from force to the motor is exponential to allow for a finer distinction at low forces. The force is updated at a frequency of 20 Hz. The transmission latency from the glove to the insole is 44 ± 3 ms which is sufficiently low for the intended application (Ismail and Shimada, 2016).

#### 2.1.2. Subunits of the system

##### 2.1.2.1. Glove

The glove was modeled especially for the iLimb quantum (Össur hf, Iceland) and can be donned on the index finger and thumb. It is made of silicone (Sili-Sil RTV-33 translucent, shore hardness A 33) and has embedded sensors (SingleTact 10 N, 8 mm) on the fingertips. The wires from the sensors to the microcontroller board are bent in waves to allow the silicone to stretch when the prosthesis is moving. Both sensor measure simultaneously. Only the higher value of the two sensors is taken as the grip force and sent from the glove to the insole.

##### 2.1.2.2. Insole

The insole is made of medical grade silicone (Silbione RTV 4428, shore hardness A 28) and can be slipped into a common house shoe. It has five embedded eccentric rotating mass motors (JinLong Machinery, diameter 10 mm, thickness 2.7 mm) that vibrate at a buzz (250 ms at 100 Hz). The motors are positioned along the foot in regions of a high density of fast adapting mechanoreceptors. There are two types of fast adapting mechanoreceptors. They are predominant on the sole of the feet with a low detection threshold at vibrations from 50 to 100 Hz (Kennedy and Inglis, 2002).

Additionally, they have small receptive fields. The insole was produced in three sizes (small, medium, large) to provide the participants with adequate systems. We defined encoding small forces at the toes and increasing force toward the heel. The microcontroller board with motor driver is placed above the fastener of the house shoe (Figure 1).

##### 2.1.2.3. Monitoring

Additionally to the sensing and feedback device, the FeetBack system may include a monitoring unit. It connects to a computer *via* USB and wirelessly to the insole. It allows surveying the sensed force with the corresponding vibrating motor on the screen.

### 2.2. Participants

Four participants (Table 1) with unilateral congenital below elbow limb absence took part in the study. All were experienced users of myoeletric prosthesis with several years of experience. Especially, all participants were familiar with the same multi-articulating prosthesis and conducted the experiments with their personal device (iLimb quantum, Össur hf, Iceland). The prosthesis was merely modified by adding the sensor glove on top of the regular cover. This setup allowed the participants to experience the feedback with a virtually unaltered internal model of the feedforward control.

**Table 1:** Demographic and clinical overview of the trial participants.

**ID**	**Gender**	**Age**	**Reason for prosth**.	**Mostly used type of prosth**.	**Daily use of prosth**.
1	M	43	Dysmelia	Myoelectric	Sometimes
2	F	41	Dysmelia	Myoelectric	Always
3	F	20	Dysmelia	Myoelectric	Sometimes
4	M	52	Dysmelia	Cosmetic	When working

The participants were asked to answer a pre-study (Table 2) and a post-study (Table 3) questionnaire. The pre-study questionnaire focused on the participant's use of the prosthesis and the expectations of sensory feedback. The post-study questionnaire focused on their impressions of the used sensory feedback.

**Table 2:** The participants' answers to the pre-study questionnaire.

**ID**	**When do you use the prosth.?**	**When do you not use the prosth.?**	**When could feedback be beneficial?**
1	Sports, presentations	When water is involved	Handshake
2	Almost everything	When water is involved	Unsure
3	Kitchen, chores, opening purses	Fine-motor tasks	When quick actions are required
4	Working, gardening, shopping	Sports, free time	Cooking, crafting

The questionnaire included open questions about the participants' personal use of the prosthetic hand and their expectations of sensory feedback.

**Table 3:** The participants' answers to the post-study questionnaire.

**ID**	**Benefit**	**What did you like about FeetBack?**	**Comments and suggestions**
1	Yes	N/A	N/A
2	No	To experience force feedback	Unsure about the benefit while other sensations, e.g., vision and audio, are available. The benefit was clear without other sensations.
3	Yes	To experience force feedback and to explore some of the fine-motoric capabilities and limits of the high-end prosthesis	N/A
4	No	Nothing in particular	Feedback at the feet seems unpractical - feedback in the socket may be a better idea. Suggestions for enhancements of the prosthesis: 1) add push buttons on the socket to quick select grasps, 2) reduce the weight of the prosthesis, 3) reduce the noise of the prosthesis, and 4) make thinner and more long living skin for the hand.

The questionnaire included open questions about their subjective impressions of the FeetBack system.

The first participant was enrolled in September 2021 and the last participant visit was in November 2021. The recruitment took place in August 2021 within the population of patients of the Balgrist University Hospital (Zürich, Switzerland). Inclusion criteria: healthy people, 18–55 years old, with basic knowledge of and trust in modern technology, and unilaterally experienced users of the iLimb quantum multi-articulating hand (independently of the cause, e.g., dysmelia or amputation). Exclusion criteria: skin incompatibilities with silicone or cognitive impairment.

### 2.3. Experiments

**Figure 2:**
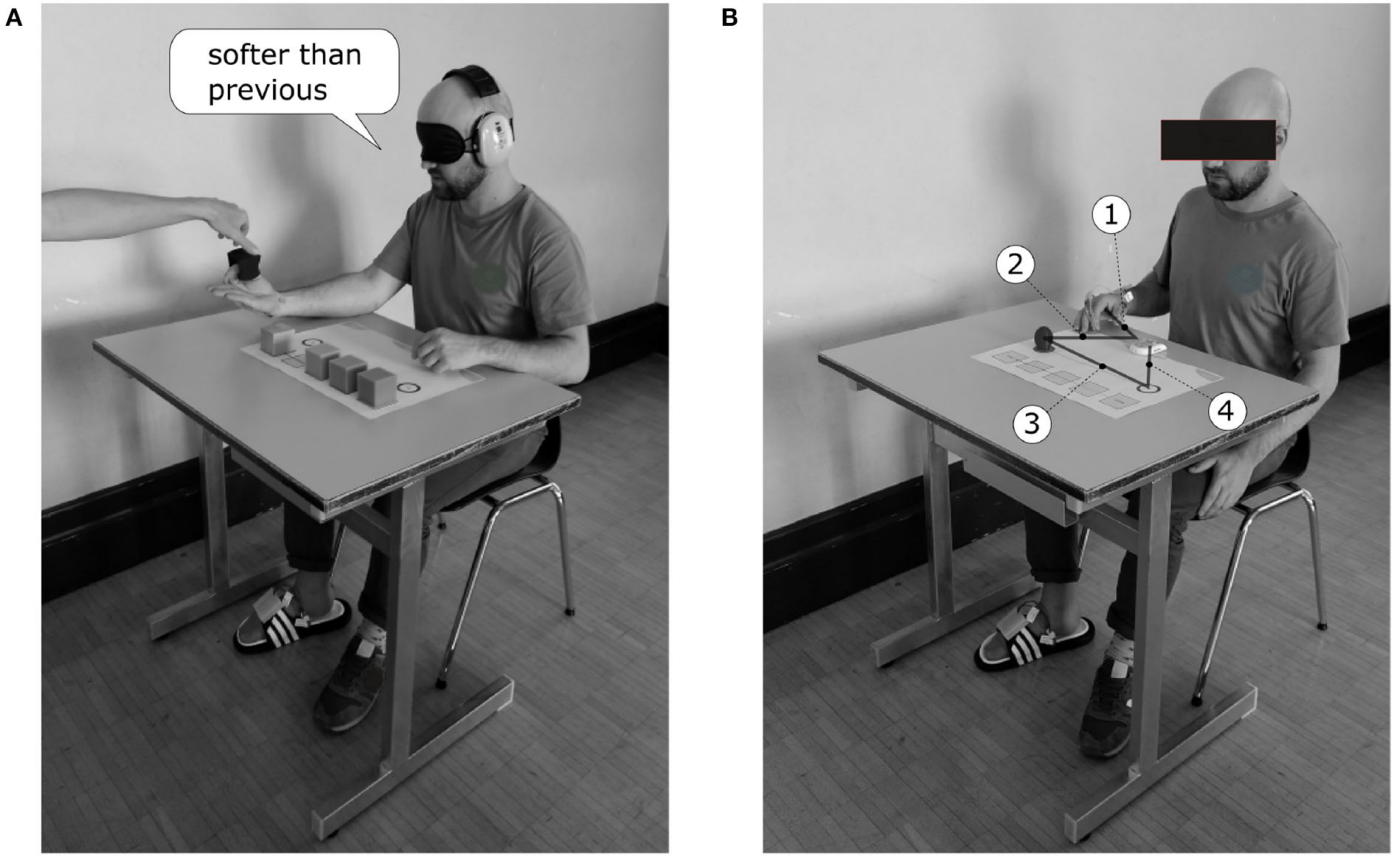
Setup of the experiments. The FeetBack glove was donned on the cover of the prosthetic hand and the FeetBack insole was worn on the right foot. The participants remained seated throughout the tests. **(A)** In the object sorting task, the participants were blindfolded and were wearing ear muffs. They rested the arm with the prosthesis on the table with the open hand upward. The investigator put the objects between the index finger and thumb. The participants had to answer if the current object was harder or softer than the previous object. **(B)** In the pick and place tasks, the participants started in a resting position as shown. After an oral start signal by the investigator, the participants moved the hand from the starting position to the clock and started said clock with the prosthesis in step 1. In step 2, the participants moved the prosthesis from the clock to the object and pinched it. In step 3, they lifted the pinched object from the side of the prosthesis to the contra-lateral target and released the object. In step 4, they stopped the clock with the prosthesis.

Four different tasks were conducted to test the applicability of force feedback from the hand to the feet. The tests varied in the availability of other feedback channels, i.e., audio-visual feedback, and in task complexity. All four tasks were conducted in two sessions of which the second followed 3–4 weeks after the first session. The participants remained seated throughout the experiments to maintain a stable sensation of the actuators (Figure 2). They were allowed to use only the thumb to index finger pinch grip for all tasks. The only exception was the object manipulation task with heavy objects. There, the participants were allowed to use the power grip due to the larger size of the object.

#### 2.3.1. Calibration and training

Prior to the tasks, the FeetBack system was explained to the participants. They were told that the force at the fingers represented the grasp force. Small forces are felt at the toes, and increasing forces are felt toward the heel, respectively. Each participant was provided the insole that had the best fit in size. The participants were asked to adjust the position of their foot on the insole such that they could perceive every actuator. Thereafter, the system was calibrated to the prosthesis of the participants. The participants were asked to squeeze the softest and hardest cube halfway three times. The force was recorded at every squeeze and the minimum and maximum force were set by the respective average.

Then, the participants were given time to learn the force feedback provided by the FeetBack system. The participants were first given 2 min to freely handle a variety of soft cubic sponges and were allowed to compare the sensation with both hands. Then, they cracked two 3D printed egg shells with the prosthetic hand to feel the allowed threshold in a later task (fragile objects). Finally, they were given two more minutes to freely handle a heavy cylinder to determine the minimum grasp force needed to lift it (although all participants noticed that they might as well just apply the full force without losing much time). After the training was completed, the tasks were conducted directly. The participants were allowed to take breaks between tasks but none made use of the offer.

#### 2.3.2. Object sorting

In one task, the participants had to sort five equal sized cubes of individual plasticity from softest to hardest without audio-visual feedback (Figure 2A). The final arrangement of the cubes was recorded as measurement. The side length of each cube was 50 mm. Four cubes were made of miscellaneous foamed plastic (weight: < 6 g) with compression load deflections of 3.0 kPa, 4.1 kPa, 5.5 kPa, 11.0 kPa, respectively. The fifth cube was made of wood (weight: 45 g).

The participants were blindfolded and wore earmuffs to eliminate audio-visual feedback. They rested their arm on a table with the open hand upside. They were given one cube at a time by the investigator. The participants were allowed to close and open the artificial hand at their chosen pace. After each cube, they had to answer if the current cube was harder or softer than the previous cube. This procedure was repeated for five runs, where one run means that all cubes were handed to the participants once. The initial order in the first round was predefined random (same order for all participants). From the second to the last round, the first given cube was always the presumably softest.

To accomplish this task, the participants had to rely on the rate of change of the force feedback and their intended sEMG signal to close the hand. This task was only performed with the feedback switched on. A comparative measurement without feedback was omitted since previous comparative tests showed that the answers were close to random guesses (Huang, 2018).

#### 2.3.3. Object manipulations

In three tasks, the participants had to manipulate fragile, heavy, and delicate objects. The success rate and time needed to accomplish a task were recorded as measurements. The success criterion depended on the specific task and will be addressed in the following paragraphs. The time was measured with a stop watch that the participants had to start before and stop after every manipulation (Figure 2B).

All object manipulation tasks were repeated 10 times with feedback and 10 times without feedback. In the first session, all tasks were conducted with feedback followed by repetitions without feedback. In the second session, the order of conduction was reversed to reduce the bias due to the learning of specific tasks through repetitions.

##### 2.3.3.1. Fragile objects

The participants had to pick and relocate an egg shell (made of 3D printed PLA, break point: 9.2 N ± 0.8 N, height: 48 mm, width: 40 mm, wall thickness 0.4 mm). The distance was 30 cm with a precision of ±1 cm. The relocation was measured as successful when the egg was placed within the fixed boundary without being cracked or dropped on the way. This task is judged to be of moderate difficulty compared to the other two manipulation tasks. The reason, therefore, is that only one hand is needed, allowing the skilled participants to rely heavily on feedforward control.

##### 2.3.3.2. Heavy objects

The participants had to pick and relocate a heavy cylinder (made of aluminum, weight: 462 g, diameter: 60 mm, height: 105 mm). The distance was 30 cm with a precision of ±1 cm. The relocation was measured as successful when the cylinder was placed within the fixed boundary without being dropped along the way. This task is judged to be the easiest of the three manipulation tasks since only one hand is needed and full force can be applied.

##### 2.3.3.3. Delicate objects

The participants had to pick a cherry and remove the stem from the body (made of modeling clay and toothpick, diameter: 16 mm, height: 24 mm, depth of toothpick: 18 mm, weight: 7.5 g). The manipulation was measured as successful when the stem was removed from the body and if the body of the cherry was not squashed [similar to Tan et al. (2014)]. This task is judged to be the most complex of the three manipulation tasks since both hands are needed. Furthermore, the right amount of grasp force must be applied to securely hold the cherry without squashing it.

### 2.4. Data analysis

The primary and secondary outcomes are defined as follows:

*Primary outcome*: Success rate to detect the contact force levels to differentiate between different objects and to manipulate various objects, using a hand prosthesis with/without tactile feedback.

*Secondary outcome*: Time needed to finish a set of manipulation tasks, using a hand prosthesis with/without tactile feedback.

#### 2.4.1. Primary outcomes

The primary outcome of the object sorting task was assessed with a measure of the ranked correlation between the participants' order and the true order from softest to hardest. We used Goodmann-Kruskal's gamma


(1) $$\begin{array}{l} G=\frac{N_{s}-N_{d}}{N_{s}+N_{d}}, \end{array}$$

where *N*_*s*_ is the number of concordant pairs and *N*_*d*_ is the number of reversed pairs. A value of *G* = 1 represents perfect order, whereas *G* = −1 represents perfect inverse order (Goodman and Kruskal, 1954).

For the object manipulation tasks, the primary outcomes were compared qualitatively within individual participants. The reason for that is that the sample size in this pilot study is too small to use quantitative methods.

#### 2.4.2. Secondary outcomes

The time needed for the manipulation tasks was modeled with linear mixed effects models. They are an extension of ordinary linear models that allow modeling fixed and random effects. The intervention (feedback turned on or off) is the fixed effect and the individual participants are random effects. This allows us to model the baseline time needed to manipulate an object depending on the participant. We used the *fitlme* method from the Statistics and Machine Learning Toolbox in MATLAB (version R2021a). The manipulation tasks with fragile and delicate objects were modeled as “time~feedback+(1∣ID)”. This formulation corresponds to an individual offset per participant and a fixed slope for the fixed effect over all participants. The manipulation task with heavy objects was modeled as “time~feedback+(feedback∣ID)”. This corresponds to an individual slope for the fixed effect per participant, since the residuals were not normally distributed with the former formulation.

## 3. Results

All participants conducted all tasks and all data was considered for the evaluation.

### 3.1. Pre-study and post-study questionnaires

The questionnaires (pre-study: Table 2; post-study: Table 3) give subjective impressions about the individual participant's expectations and impressions of force feedback. The expectations before the trials show that three out of four participants see potential situational benefits of adding force feedback to prosthetic hands. However, ID 2 who reportedly uses her prosthesis the most (“always,” “[for] almost everything”), was unsure about the benefits. The impressions after the trials changed the point of view for ID 4 and remained the same for the other participants. Participants ID 1 and ID 3 were believed to experience a benefit from FeetBack, although with limited excitement. Participant ID 2 repeated her prior opinion and observed a few advantages of force feedback under the presence of visual-audio feedback. Participant ID 4 saw no benefit in force feedback specifically at the feet. Furthermore, ID 4 suggested several improvements on the multi-articulating prosthetic hand as it is before adding force feedback.

### 3.2. Experiments

#### 3.2.1. Object sorting task

**Figure 3:**
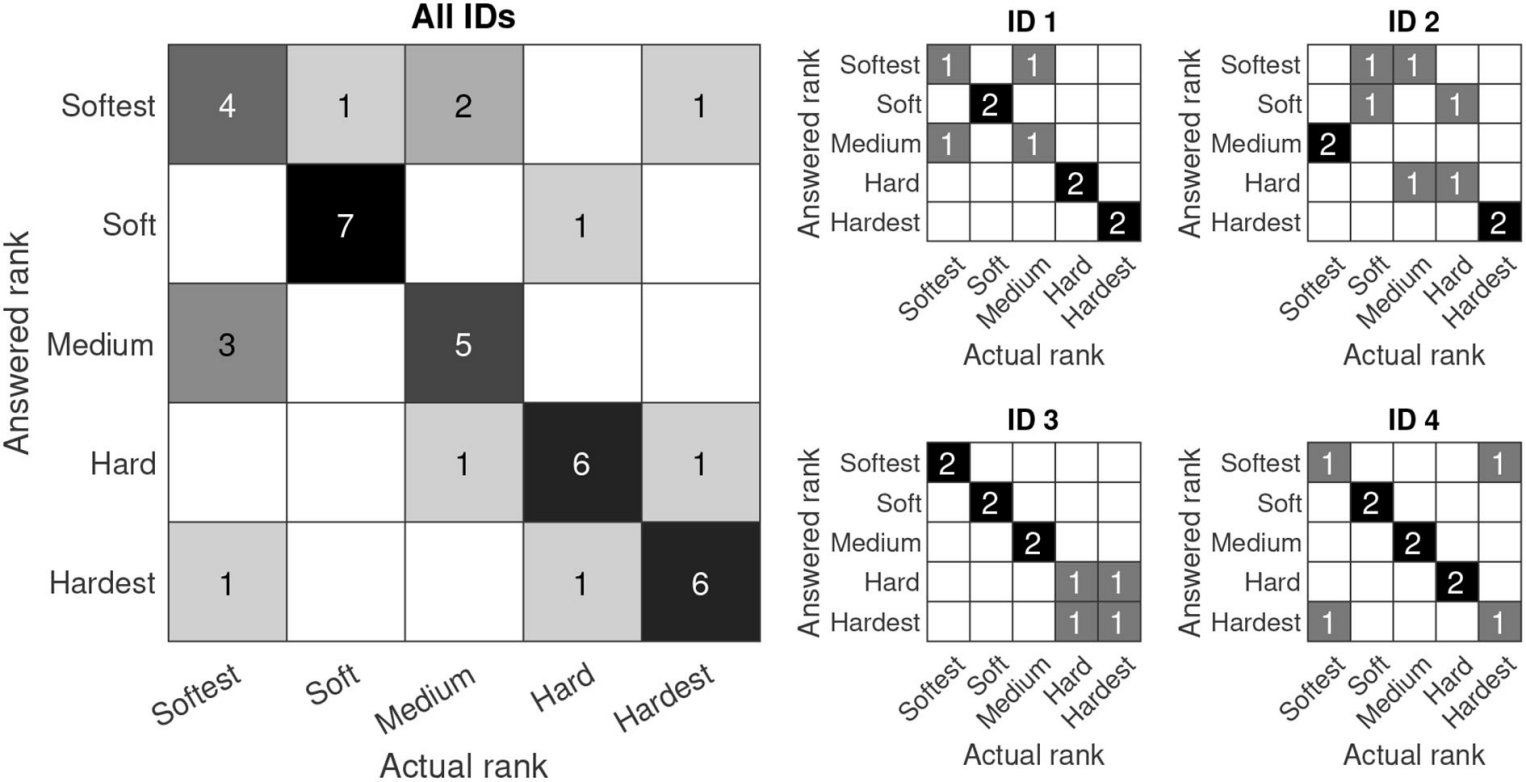
Confusion matrix of the object sorting task with feedback. In the ideal case, all answers would lie in the diagonal of the confusion matrix. Since the grading is ordinal, errors become more severe the farther they are from the diagonal. (left) Cumulative matrix with observations from all participants; (right) Individual observations per participant.

Goodmann-Kruskal's gamma was *G* = 0.639, suggesting that feedback has a substantial positive effect. Three out of four participants merely confused two objects (Figure 3), meaning they all had one perfect sorting session out of two. Participant ID 2 confused two objects in one session ('*softest*' and '*medium*') and multiple objects in the other session.

#### 3.2.2. Object manipulation tasks

##### 3.2.2.1. Success rates

**Figure 4:**
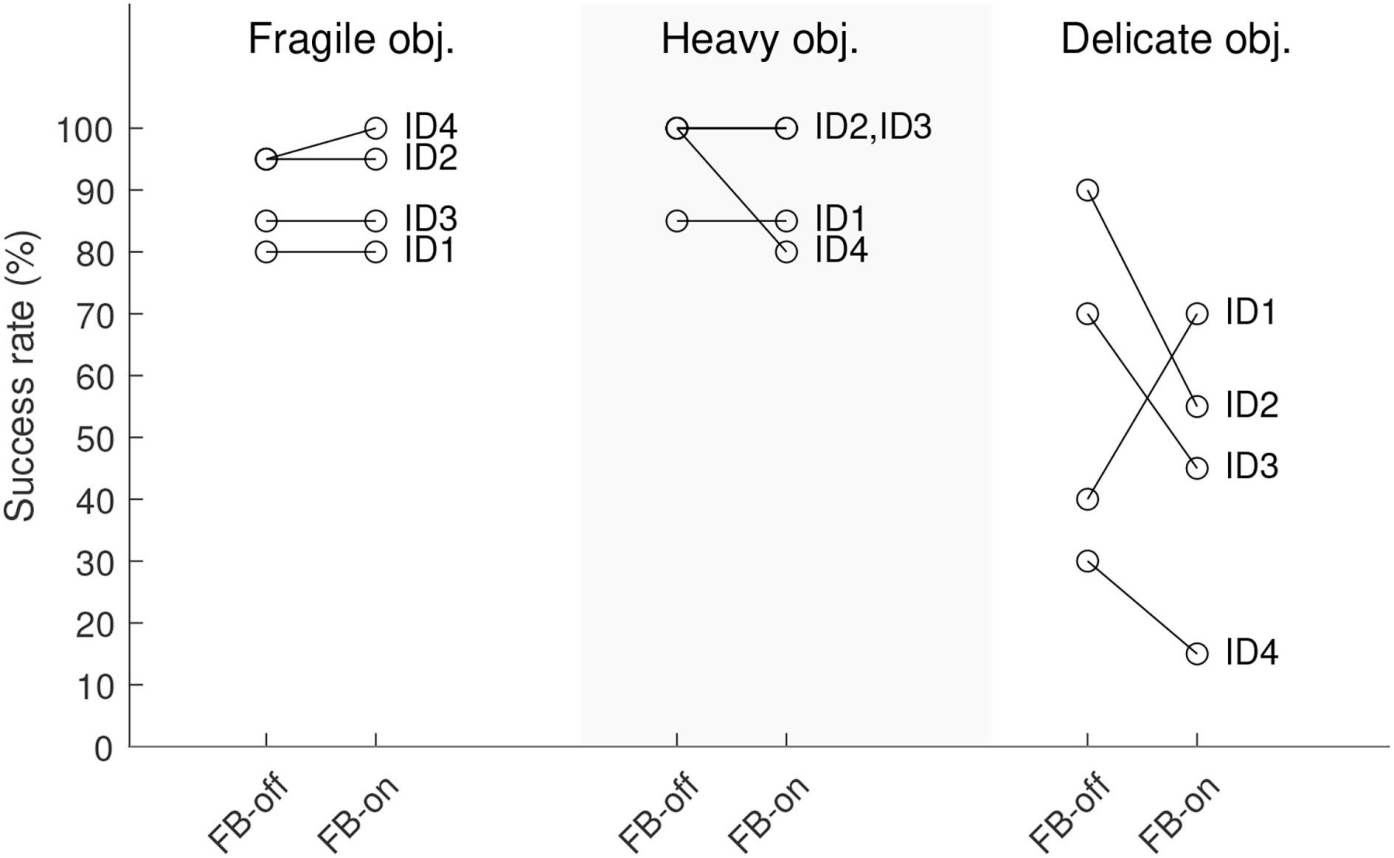
Success rates of the object manipulation tasks. All participants performed n=20 manipulations per task under each condition FB-on (feedback switched on) and FB-off (feedback switched off).

In the single-handed tasks with fragile and heavy objects, the success rates were high (SR ≥80%, Figure 4) for all participants regardless of the intervention (feedback switched on or off, respectively). In the two-handed task with delicate objects, however, the success rates were generally lower and varied between participants. Three participants performed worse when force feedback was provided.

##### 3.2.2.2. Time needed to perform task

There is a positive effect (Table 4) in the tasks with fragile objects (4.209 s+0.326 s, baseline intercept + feedback) and the delicate objects (3.900 s+0.406 s, baseline intercept + feedback). The 95 % CI does not encompass 0.0 s in both cases. This suggests that there was a significant increase in time needed to complete these two manipulation tasks with feedback. There is no significant effect of the feedback in the task with heavy objects as the 95 % CI clearly encompasses 0.0 s (Table 4). Residuals of the models were normally distributed.

**Table 4:** Linear mixed effect model of the time needed for manipulation tasks.

**Manipulation**	**Fixed effect**	**β**	**95 % CI**	**Participant (SD)**	**Corr**
Fragile obj.	Intercept	4.209	[3.514, 4.904]	0.677	N/A
	Feedback	0.326	[0.058, 0.593]	N/A	
Heavy obj.	Intercept	4.242	[3.310, 5.173]	0.932	0.031
	Feedback	–0.019	[–0.551, 0.513]	0.499	
Delicate obj.	Intercept	3.900	[3.017, 4.783]	0.860	N/A
	Feedback	0.406	[0.087, 0.725]	N/A	

The estimated fixed effects (intercept, feedback) and random effect (participant ID) with the corresponding 95% confidence interval (CI) and standard deviation (SD) of the random effect are presented for every experiment individually. Since the experiment with heavy objects was modeled with an individual slope per participant, the correlation between the fixed effects is given, too.

## 4. Discussion

Initially, the limitations of the study are discussed to put the results into context. The results are discussed according to the small number of research participants in this pilot study. Furthermore, the study design only allowed us to explore the *immediate* effect of vibrotactile feedback at the feet since the participants merely used the system twice for a couple of hours. Finally, we deliberately chose to simulate the user case scenarios that incorporate the internal model of the user in the interaction with their prosthesis. Although our approach allows for a holistic scenario with human-machine interaction, it is more challenging to isolate confounding factors; namely the accuracy of the feedforward control of the prosthesis and the incidental feedback. In fact, recent research (Gholinezhad et al., 2021) proposed a method to first assess the effects of the feedback on the natural hand against natural feedback. The benefit of the feedback may then be tested with active users of prosthetic devices once the effects are estimated. Nevertheless, we believe that this pilot trial toward a novel feedback approach resulted in valuable insights for future investigations.

The information of the vibrating insole was interpreted successfully when audio-visual feedback was prevented, as demonstrated in the sorting task. This shows that discrete vibrotactile feedback at the feet can indeed be used to translate information about the grasp force. However, participants ID 2 and ID 3 reported feeling rather unsure about their answers. At this stage, it is not possible to pin the exact causes. Although, the likely reasons can be the short time the participants had to learn to interpret the feedback and inadequate implementation of the feedback device in terms of resolution and perceptibility.

The success rates during manipulation tasks were overall equivalent or even lower when vibrotactile feedback at the feet was provided. The outcomes from the pick and place tasks (fragile and heavy objects) were not altered noticeably by the intervention. In the task with heavy objects, this was anticipated, since the participants could merely use full force. However, the equivalent success rate for the task with fragile objects was unexpected. An exhaustive review on feedback strategies for prosthetic hands by Sensinger and Dosen (2020) provides possible reasons when feedback would not improve the success rate; namely, an already efficient internal model, sufficient incidental feedback, a task that is too simple, or a weak feedback method. We believe that the most dominant causes are the efficient internal models coupled with a rather simple task in which the experienced users did not rely on additional feedback (other than audio-visual and incidental feedback). The review by Jabban et al. (2022) supports the notion that object manipulation tasks with fragile objects may underestimate the benefit of sensory feedback. The reason for this is that such tasks can be solved by feedforward control alone.

In the most complex manipulation task with delicate objects, the success rate was considerably lower when feedback was provided. Unlike Tan et al. (2014), our approach to *the cherry stem removal task* resulted in overall inferior success. The tests are not directly comparable since one uses actual cherries and the other uses replica made of modeling clay and toothpicks. Nevertheless, we assume that our approach reached inferior success rates due to an insufficient resolution of the discrete spatial coding. In the case of participant ID 4 who reportedly tried to incorporate the feedback for fine-tuning, the interaction of human, prosthesis, and FeetBack resulted in fluttering pinches that squashed most of the fake cherries. Thus, the presented system does not allow for fine-tuning the grip for sophisticated closed-loop control. However, it serves to notify the user about initial contact and may help engage the user. This was likely the case for participant ID 1 who reportedly perceived a subjective benefit.

The increase of time needed for manipulation tasks with fragile and delicate objects could be explained by the new sensation of vibrotactile feedback, cognitive burdening, or a poorly chosen location to apply the feedback. According to participant ID 4, the feet may be a bad site which potentially distracts the user. Although, the remaining three participants did not comment on that notion. Wells et al. (2022) reported similar findings, where the mechanotactile feedback resulted in an increased time to finish a task. The respective authors believe this effect to be due to the added focus on the task with feedback. Such an increase in time was not observed in comparable studies that applied discrete feedback to the residual limb (Aboseria et al., 2018; Raveh et al., 2018). However, Clemente et al. (2016) who investigated the effect of feedback for object contact and release did not observe a speed boost with feedback in a long at home trial, either. Nevertheless, their success rate to manipulate fragile objects increased. Therefore, they reason that feedback may be costlier from a cognitive perspective but allows for more confidence. In our case, participant ID 1 appears to have had this experience, too.

No participant reported an adverse effect due to the feedback method. Nevertheless, a potential adverse effect of feedback applied to the feet could be *sensory steering* (Zehr et al., 2014). In that case, electrotactile stimulation at the sole of the feet provoked potentially unwanted muscle activities. Velázquez et al. (2012) also applied vibrotactile feedback in a walking navigation task and they did not encounter any such undesired effect. We did not investigate the effect of FeetBack in standing or walking tasks. However, we expect that our system would behave similarly to the system presented by Meier et al. (2015) where an increasing walking speed affects the perceptibility negatively. The reason therefore is that the foot may momentarily loose contact to the insole or exert too much pressure on the motors. However, such mechanical issues would need to be addressed in the next iteration of FeetBack. Also, according to a survey with 142 unique responses (Smither et al., In Press) the most anticipated benefits were reported in stationary tasks such as zipping jackets, tying shoes, buttoning shirts, and using a cup.

In summary, we observed that the proposed feedback method causes various effects depending on the tasks. Without audio-visual feedback, the gain was evident as the participants were generally able to distinguish between objects of distinct plasticity. However, in the more realistic setting with vision and hearing the anticipated benefit was not achieved. Similar observations were made by Markovic and Schweisfurth (2018) where an advanced feedback method was only helpful in complex tasks for regular users of hand prostheses. The easier tasks could be learned accurately by feedforward control after repeated executions. Thus, we further support the notion that the study design for experienced users of upper limb prostheses must be more complex in order to assess the feedback method's capacity and impact on the users (Sensinger and Dosen, 2020; Jabban et al., 2022).

## 5. Conclusion

The viability of vibrotactile feedback in (mobile) robot control and navigation tasks had already been shown. We anticipated investigating its potential within the field of hand prostheses. We showed for the first time that information from the prosthetic hand can be interpreted at the feet with beneficial effects when sight and vision are prevented. The results suggest that the immediate effect of discrete spatially coded vibrotactile feedback at the feet allows distinguishing between plastic objects without the help of vision and hearing. However, the interaction of human, myoelectric hand, and FeetBack does not allow the grasp force to be qualitatively fine-tuned under pressure of time. Moreover, there appears to be little benefit from FeetBack under the presence of audio-visual feedback for experienced users of myoelectric hands. These findings are in line with findings of similar studies with tactile feedback provided on the arm or within the prosthetic socket. Hence, we provide a rationale to further investigate the clinical benefit of feedback redirected to the feet in a large-scale clinical trial. Ultimately, we suggest testing the clinical benefit of such feedback not only under laboratory conditions alone but also in an at-home trial over a longer period of time.

## Data availability statement

The datasets generated for this study are available on request to the corresponding author.

## Ethics statement

The studies involving human participants were reviewed and approved by Cantonal Ethics Committee in Zürich, Switzerland (Ref. No.: 2019-01639). All experiments were conducted in accordance with the declaration of Helsinki, and all participants provided written informed consent prior to participation in the experiments.

## Author contributions

RM, NS, TB, and VK participated in the design of the study. MB screened the participants. RM, NS, and SC conducted the tests. RM, NS, and TB analyzed the data. RM wrote the manuscript. TB, NS, MB, SC, and VK revised and approved the manuscript. All authors contributed to the article and approved the submitted version.

## Funding

This study was supported by the foundation Inventus Bern, Switzerland (Grant No. 39/2019).

## Conflict of interest

The authors declare that the research was conducted in the absence of any commercial or financial relationships that could be construed as a potential conflict of interest.

## Publisher's note

All claims expressed in this article are solely those of the authors and do not necessarily represent those of their affiliated organizations, or those of the publisher, the editors and the reviewers. Any product that may be evaluated in this article, or claim that may be made by its manufacturer, is not guaranteed or endorsed by the publisher.

## Footnotes

^1^ https://bionicsforeveryone.com/current-options-for-bionic-hands
